# Mortality in the German Pharmacoepidemiological Research Database (GePaRD) compared to national data in Germany: results from a validation study

**DOI:** 10.1186/s12889-015-1943-7

**Published:** 2015-06-20

**Authors:** Christoph Ohlmeier, Ingo Langner, Kathrin Hillebrand, Niklas Schmedt, Rafael Mikolajczyk, Oliver Riedel, Edeltraut Garbe

**Affiliations:** Leibniz-Institute for Prevention Research and Epidemiology – BIPS, Achterstr. 30, 28359 Bremen, Germany; Hubertus Wald Tumor Center - University Cancer Center Hamburg (UCCH)/University Medical Center Hamburg-Eppendorf (UKE), Martinistr. 52, 20246 Hamburg, Germany; Helmholtz Centre for Infection Research, Inhoffestr. 7, 38124 Braunschweig, Germany; Hannover Medical School, Carl-Neuberg-Str. 1, 30625 Hannover, Germany; Core Scientific Area ‘Health Sciences’ at the University of Bremen, Grazer Str. 2, 28334 Bremen, Germany; Department of Human and Health Sciences, University of Bremen, Grazer Str. 2, 28334 Bremen, Germany

**Keywords:** Administrative data, Claims data, Health insurance data, Mortality, Death, Validation, Germany

## Abstract

**Background:**

Electronic healthcare databases are of increasing importance in health research and mortality is one of the most relevant outcomes. However, data in these databases need to be validated, since they are often generated for reimbursement purposes. The aims of this study were to compare mortality figures from the German Pharmacoepidemiological Research Database (GePaRD) on an aggregated level with external data from the Federal Statistical Office of Germany (FSOG) and to assess consistency of records of death from core data and hospital data within GePaRD.

**Methods:**

The study population comprised insurants of four statutory health insurances providing data for GePaRD with either continuous insurance coverage from January 1^st^ to December 31^st^ 2006 or until death. The sex-specific mortality rate, stratified and standardized by age, and the percentage of hospital deaths among all deaths was compared with data from the FSOG. Furthermore, the agreement between the dates of death according to hospital data and core data was assessed within GePaRD.

**Results:**

The study population comprised 12,033,622 insurants. Compared to FSOG data, the age-standardised mortality rate in GePaRD was 21 % and 29 % lower in women and men, respectively. Regional analyses also indicated lower mortality rates in all federal states except for Bremen, where the age-standardised mortality rate was similar to FSOG data for both sexes. The percentage of hospital deaths among all deaths corresponded well with external data. The proportion of inpatient deaths also recorded in the health insurance core data was 98.5 %. Furthermore, 94 % of dates of death documented in hospital agreed with the dates of death according to the health insurance core data.

**Conclusions:**

The lower mortality rates in almost all federal states might result from the higher socioeconomic status of the GePaRD study population compared to the overall population in Germany. In the federal state of Bremen, where socioeconomic representativeness is higher due to additional inclusion of two local health insurances, the mortality rates were in good accordance with external data. Agreement of the percentage of hospital deaths among all deaths between GePaRD and national statistics suggested completeness of outpatient mortality information.

## Background

Large healthcare databases are increasingly being used in the field of health services research, providing an unbiased insight into routine care [1]. However, since data in these databases are generated mainly for documentation and reimbursement purposes and not for research purposes, they need to be validated [2]. One of the most essential information in health research is mortality. It is of most importance when evaluating the benefit, risk or quality of care regarding (new) drugs or other interventions or health care services. Furthermore, mortality is also important for the statistical analyses as it can lead to censoring in Kaplan Meier analyses.

In data of German statutory health insurances (SHIs), a death is documented in the core data as a potential reason for leaving the insurance company. For persons dying in hospital, a death is additionally documented in the hospital data. While the date of death in hospital data is based on reports from the hospital, in core data it is based on notifications of the insurance company by other sources.

Information of administrative databases can ultimately only be validated based on a record linkage with another data source containing the gold standard information [3]. However, this is associated with very high data protection requirements in Germany and also very expensive and time-consuming. Therefore a record linkage can often not be realised. In this context, a comparison of the relevant aggregated information, e.g. the mortality rate in this study, with a national statistic can give first insights into the completeness and validity of the database.

The aims of this study were to compare mortality figures of the German Pharmacoepidemiological Research Database (GePaRD) with external data from the Federal Statistical Office of Germany (FSOG) on an aggregated level. In contrast, in a second step, we compared two sources of information regarding the date of death within GePaRD on an individual level.

## Methods

### Data source

Source of data for this study was GePaRD that has been built by the Leibniz Institute for Prevention Research and Epidemiology – BIPS [4]. GePaRD consists of claims data from four German SHIs which included data of more than twelve million persons in 2006 from all geographical regions of Germany. Membership in an SHI is compulsory in Germany for employees below an annual income threshold (47,250€ in 2006). Subjects with higher incomes can choose private health insurance providers instead of an SHI and are somewhat underrepresented in SHIs [4, 5]. However, a considerable proportion of these persons remain voluntary members of SHIs, most often because SHIs provide free health insurance for unemployed family members (children and spouse) whereas in private health insurance plans all family members have to be paid for. About 70 million people (85 % of the German population) are SHI members, including about five million voluntary members, children and patients who are retired or unemployed. On the other hand, there may also be some overrepresentation of patients with middle to higher socioeconomic status in GePaRD, since three of the four SHIs contributing to the database are more likely to insure patients of middle to higher socioeconomic status. However, the database also includes patients from one local insurance company which traditionally insures patients of lower socioeconomic status [6].

Two large SHIs contributing to GePaRD cover all geographical regions of Germany. Two smaller SHIs mainly include insurants from Bremen and Lower Saxony. The database comprises information on demographic characteristics of the health insurance members, information on their outpatient physician contacts, hospital admissions and outpatient prescriptions.

### Inclusion and exclusion criteria

The study population consisted of insurants with either a continuous insurance period from January 1^st^ to December 31^st^ 2006 or to a documented date of death in this year. Children born in the given year were included in the study population if they were continuously insured until December 31^st^ 2006 or until a documented date of death in this year. Insurants without valid information on sex, year of birth, or region of residence were excluded from the study population. Co-insured family members of one small SHI were excluded from the study population, since the cause for the end of the insurance period in the core data was not specified for this group.

### Identification of deaths and the date of death in GePaRD

Deaths in GePaRD were identified if the reason for the end of the insurance period or the reason for discharge from hospital was “death”. The date of death was defined as the end date of the insurance period which indicated death and, for hospital deaths, the end date of the hospital stay which ended with death.

### External comparison data

To compare the mortality indicators calculated in our study with external data, the respective mortality indicators were obtained from the FSOG as reference information [7]. In Germany, the reporting of death certificates to the FSOG is regulated by law. First, deaths have to be reported to the registry office, where deaths are registered and certified. The registry offices forward the information to the regional statistical offices as well as to the health authority. Afterwards, the mortality information of the different regional statistical offices is collected at the FSOG.

### Statistical analysis

The annual mortality rate per 100,000 persons was calculated stratified by sex, age and federal state by dividing the number of deaths by the number of insurants of the respective stratum. 95 %-confidence intervals (CIs) for mortality rates were calculated by the substitution method, assuming that the number of events is Poisson distributed [8]. In order to adjust for age differences between the GePaRD study population and the population distribution of Germany, mortality rates estimated in GePaRD were age-standardised for men and women and compared to the respective age-standardised mortality rate from the FSOG. CIs for age-standardised rates were calculated according to the gamma distribution [9]. Furthermore, the percentage of hospital deaths among all deaths was calculated stratified by sex and age. As measures of internal consistency, the proportion of deaths documented by the hospitals which were also recorded as deceased persons according to the health insurance core data was calculated and the difference between the date of death obtained from the hospital data and the date of death obtained from the core data were calculated.

All analyses were performed using SAS 9.1 (SAS Institute, Inc, Cary NC).

### Ethics and legal regulations

Use of the data for research purposes was approved by the contributing SHIs and by local and federal government authorities. In accordance with §75 of volume 10 of the German Social Insurance Code, informed consent of the insurants was not required. Since the study was based on routinely collected pseudonymized data and persons were not contacted, ethical approval was not needed.

## Results

**Fig. 1:**
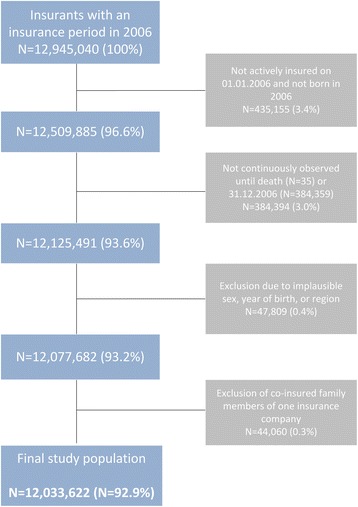
Size of the study population selected from GePaRD when applying the in- and exclusion criteria

**Fig. 2:**
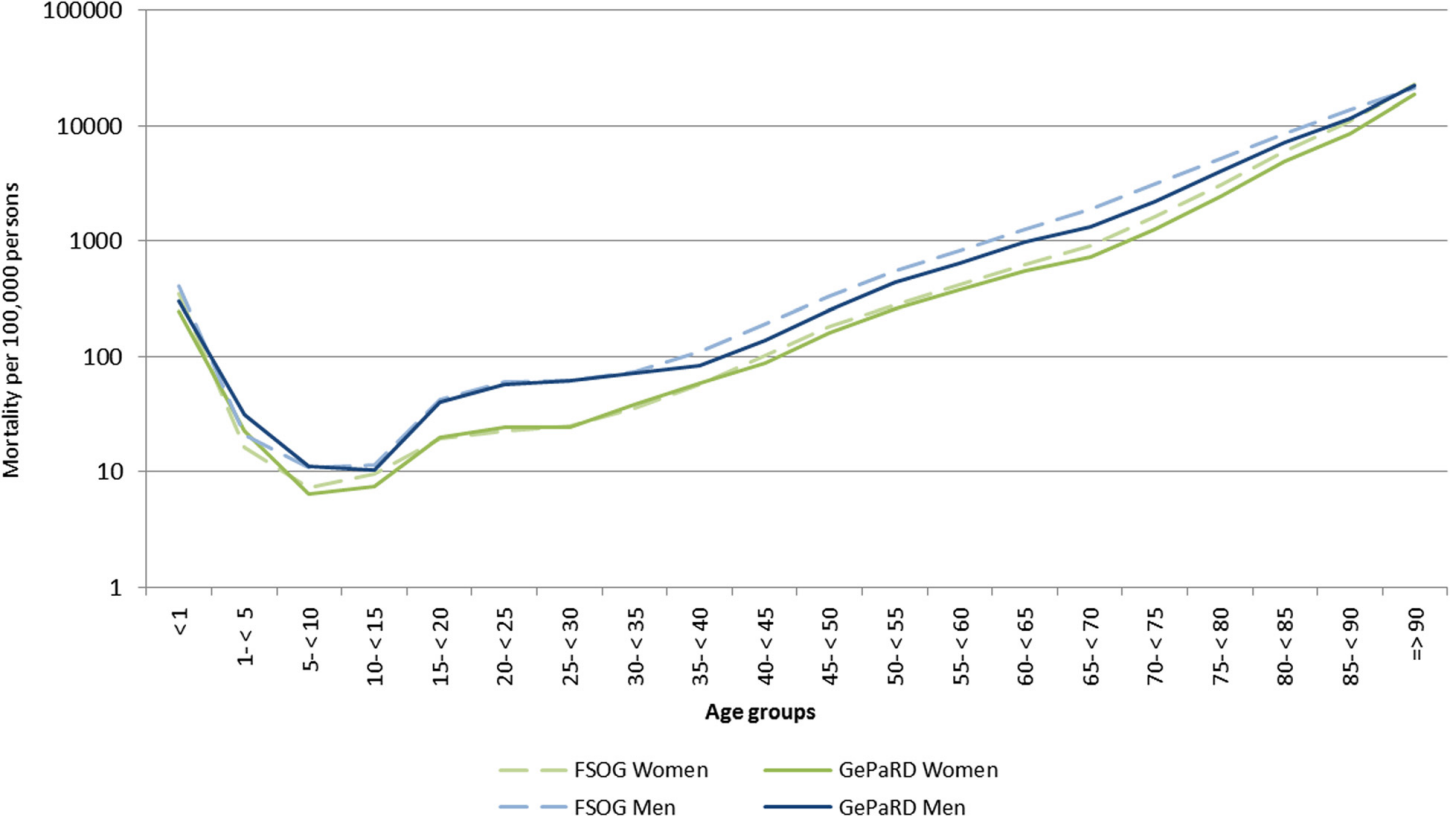
Age- and sex-stratified mortality per 100,000 persons in Germany and in GePaRD in 2006 on a logarithmic scale. FSOG: Federal Statistical Office of Germany. GePaRD: German Pharmacoepidemiological Research Database

**Fig. 3:**
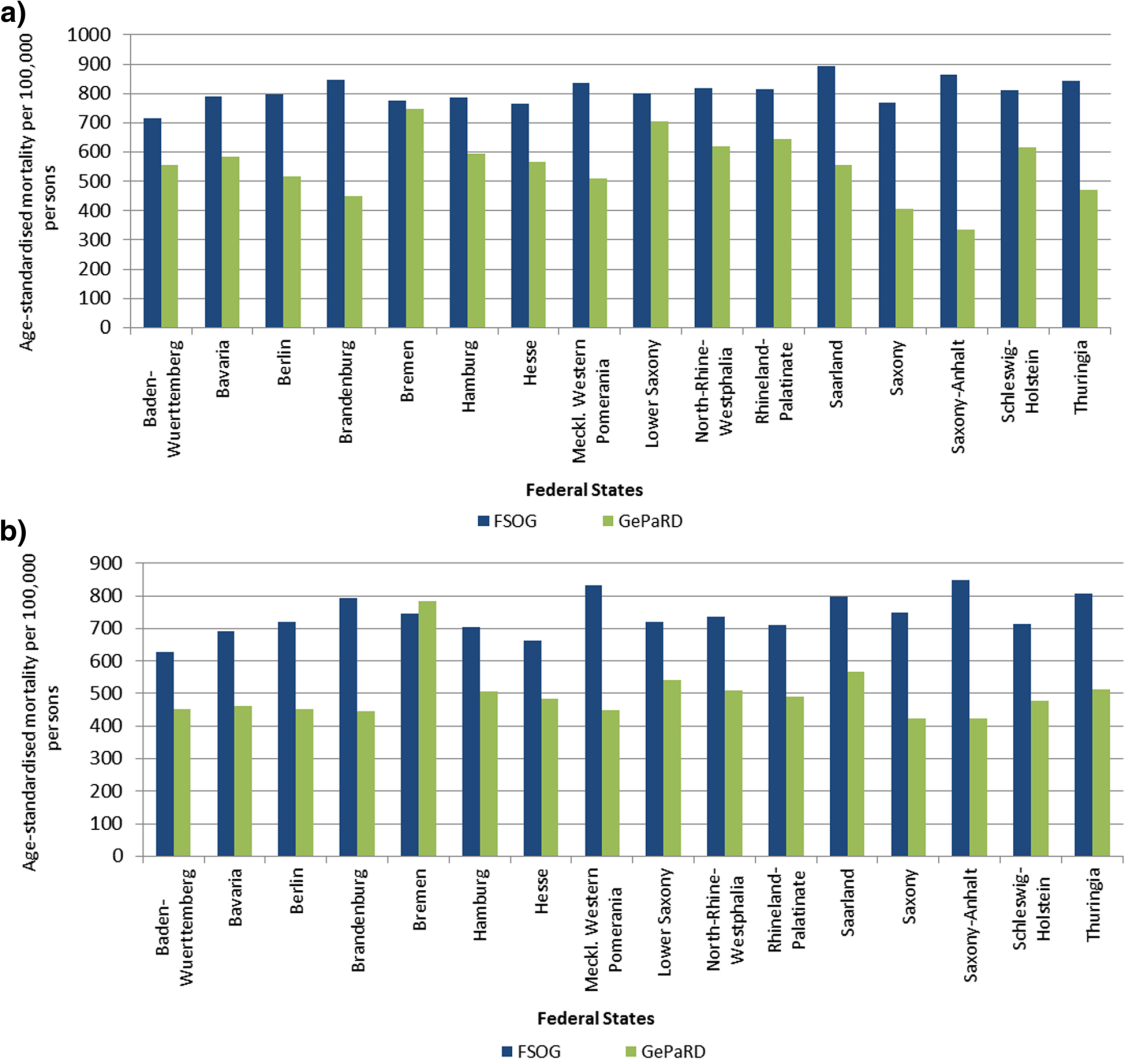
Age-standardised mortality per 100,000 persons in Germany and in GePaRD stratified by federal state in 2006 for **a** women and **b** men. FSOG: Federal Statistical Office of Germany. GePaRD: German Pharmacoepidemiological Research Database

**Fig. 4:**
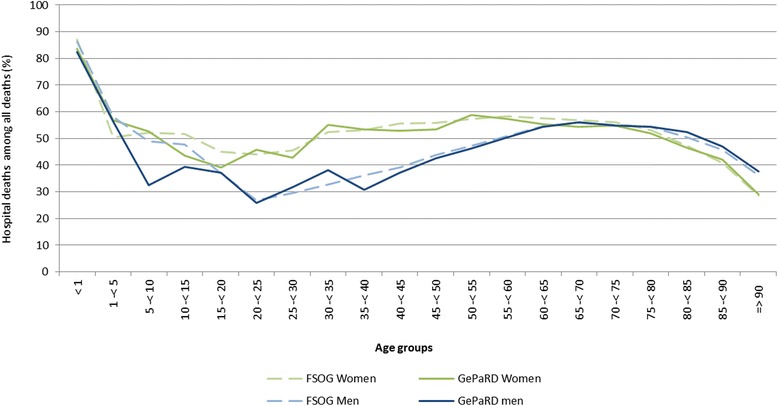
Age- and sex-stratified percentage of hospital deaths among all deaths in Germany and in GePaRD in 2006. FSOG: Federal Statistical Office of Germany. GePaRD: German Pharmacoepidemiological Research Database

The influence of the in- and exclusion criteria on the size of the study population is shown in the sample flow chart (Fig. 1). The study population comprised 12,033,622 persons of whom 44.9 % were male (Table 1). The age-standardised mortality rate in GePaRD in 2006 was 621.9 per 100,000 persons (95 % CI: 609.5-634.6) for women and 506.7 per 100,000 persons (95 % CI: 498.4-515.1) for men. These rates were 21 % and 29 % lower for women and men, respectively, compared to the data from FSOG (data not shown). The age-stratified mortality rates showed an increase with age in a similar fashion as the national data, however, somewhat lower estimates for persons in GePaRD were evident in patients aged 55 years or older (Fig. 2). Also sex-stratified standardised mortality rates by federal state showed lower mortality estimates than the FSOG in all federal states except Bremen, where the mortality rate was in good accordance with the data of the FSOG (Fig. 3 a and b). The age-stratified percentage of hospital deaths among all deaths was high in children aged younger than five years, decreased afterwards and rose again to 47-55 % in persons aged 60–85 years (Fig. 4). These results compared well with the data of the FSOG in both sexes, especially in persons aged older than 40 years. The percentage of hospital deaths among all deaths was 45.8 % in women and 50.7 % in men and was also in good accordance with the data of the FSOG, where a percentage of hospital deaths among all deaths of 44.7 % in women and 50.5 % in men were observed.

**Table 1:** Age and sex distribution of the study population in GePaRD in 2006

	Women		Men		All	
	*n*	*%*	*n*	*%*	*n*	*%*
Age group						
0-39 years	2,703,519	40.8	2,445,760	45.3	5,149,279	42.9
40-49 years	1,186,434	17.9	899,125	16.6	2,085,559	17.3
50-59 years	958,088	14.5	748,431	13.9	1,706,519	14.2
60-69 years	928,988	14.0	746,414	13.8	1,675,402	13.9
70-79 years	525,294	7.9	422,678	7.8	947,972	7.9
80-89 years	278,391	4.2	127,583	2.4	405,974	3.4
≥90 years	48,396	0.7	14,521	0.3	62,917	0.5
All	6,629,110	100	5,404,512	100^a^	12,033,622	100^a^
Mean age (Mean, SD)	43.7	22.2	40.8	22.4	42.4	22.3

^a^Percentages do not sum up to 100 % due to rounding

Almost 99 % of the deaths recorded in the hospital data were also recorded in the core data of the health insurances. The date of death in the hospital data was concordant with the date of death in the core data in 94.2 % of all deaths (Table 2). Deviations in the date of death were mostly equal or below 5 days and involved more than 5 days in only 0.1 % of all cases.

**Table 2:** Agreement between the date of death recorded in hospital data and the date of death recorded in core data in GePaRD in 2006

Difference	*n* = 42,188	
	*n*	*%*
−1 to −5 days	2	0.00
No difference	39,757	94.24
+1 to +5 days	2,389	5.66
+6 to +10 days	19	0.05
+11 to +20 days	8	0.02
+21 to +50 days	11	0.03
> +50 days	2	0.00

## Discussion

Our comparison of age-standardised mortality rates in GePaRD with national data for Germany showed a somewhat lower mortality rate for both sexes. This was also replicated in regional analyses by federal state except for the state of Bremen. The percentage of hospital deaths among all deaths was in good accordance with external data. Furthermore, a high internal consistency of the records of deaths was observed.

We assume that the lower estimates of the age-standardised mortality in GePaRD result from the higher socioeconomic status of the GePaRD study population compared to the general population in Germany. The socioeconomic status is known to be an important predictor for morbidity and mortality [10, 11]. In GePaRD, insurants with middle to higher socioeconomic status were overrepresented compared to the general population, since more than 90 % of the total study population were insured by two SHIs which traditionally insure people with middle to higher socioeconomic status [6]. An exception is the federal state of Bremen, where the SHI distribution in the study population was more balanced due to the inclusion of two local insurance companies, one of which traditionally insures people with a low or middle socioeconomic status [6]. In Bremen, the age-standardised mortality rates compared well with national statistics, supporting the assumption of socioeconomic status differences as an explanation of the lower mortality rates observed in GePaRD.

With regard to regional variations, deviations of age-standardised mortality rates between the GePaRD study population and the general population were more pronounced in the federal states of the former German Democratic Republic compared to the other federal states. After the German reunification, the population of the former German Democratic Republic, previously covered by state insurance, was enrolled in the system of SHIs existing in the former Federal Republic of Germany. While the local SHIs enrolled most of the population, presumably predominantly younger and healthier insurants switched to alternative SHIs, among them to those which traditionally insure people with middle to higher socioeconomic status. Studies suggest that persons who change the health insurance company are younger, healthier and have a higher socioeconomic status compared to those who do not change the health insurance company [12]. This selection mechanism could have been even stronger in the former German Democratic Republic, which would explain the more pronounced differences between the GePaRD study population and the general population in these federal states. Furthermore, the coverage of the population in these federal states by GePaRD is comparatively low, which also might have contributed to the more pronounced differences.

The percentage of hospital deaths among all deaths agreed well with national data in all age groups. Especially in the older age groups, the percentages of hospital deaths in GePaRD were similar to the national data, whereas the small deviations observed in the younger age groups are probably due to fluctuations resulting from the overall small number of deaths in these younger age groups. Given the fact that hospital data are likely to provide complete information about deaths, the similar percentage of patients dying in the hospital compared to national data suggests that also records of outpatient mortality are rather complete in GePaRD. However, an undercoding of deaths, which could also be one of the reasons for the lower mortality rates observed in this study, cannot be ruled out.

### Strength and limitations

The main strength of this study is the large sample which allowed robust estimations of mortality indicators. Since information on the socioeconomic status is rather incomplete in administrative data and mortality estimates stratified by socioeconomic status are lacking in the data of the FSOG, an adjustment for the socioeconomic status could not be carried out. Direct external validation of mortality associated information by medical chart review was not possible within the framework of this study.

## Conclusions

The mortality estimates were overall in good accordance with external data. The observed differences were expected due to a higher socioeconomic status of our study population. Comparison of mortality related information in core and hospital data of the database demonstrated a high consistency. The percentage of hospital deaths among all deaths in GePaRD agreed well with national statistics, indirectly also providing evidence for completeness of outpatient mortality information. Therefore, this study provides first evidence for complete and valid mortality related information in GePaRD. However, studies based on a record linkage with a data source containing the gold standard are needed to further elucidate the validity of the respective information.

## Abbreviations

SHI: Statutory health insurance
GePaRD: German Pharmacoepidemiological Research Database
FSOG: Federal Statistical Office of Germany
CI: Confidence interval
